# Incomplete insertion of pedicle screws triggers a higher biomechanical risk of screw loosening: mechanical tests and corresponding numerical simulations

**DOI:** 10.3389/fbioe.2023.1282512

**Published:** 2024-01-08

**Authors:** Jie-Xiang Yang, Lin Luo, Jin-Hui Liu, Nan Wang, Zhi-Peng Xi, Jing-Chi Li

**Affiliations:** ^1^ Department of Orthopedics, The Affiliated Traditional Chinese Medicine Hospital, Southwest Medical University, Luzhou, China; ^2^ Luzhou Key Laboratory of Orthopedic Disorders, Southwest Medical University, Luzhou, Sichuan, China; ^3^ Sichuan Provincial Laboratory of Orthopaedic Engineering, Department of Bone and Joint Surgery, Affiliated Hospital of Southwest Medical University, Luzhou, Sichuan, China; ^4^ Department of Orthopedics, Affiliated Hospital of Integrated Traditional Chinese and Western Medicine, Nanjing University of Chinese Medicine, Nanjing, Jiangsu, China

**Keywords:** screw loosening, incomplete thread insertion, biomechanical deterioration, pedicle screw, comprehensive biomechanical research

## Abstract

Screw loosening is a widely reported issue after spinal screw fixation and triggers several complications. Biomechanical deterioration initially causes screw loosening. Studies have shown that incomplete insertion of pedicle screws increases the risk of screw breakage by deteriorating the local mechanical environment. However, whether this change has a biomechanical effect on the risk of screw loosening has not been determined. This study conducted comprehensive biomechanical research using polyurethane foam mechanical tests and corresponding numerical simulations to verify this topic. Pedicle screw-fixed polyurethane foam models with screws with four different insertion depths were constructed, and the screw anchoring ability of different models was verified by toggle tests with alternating and constant loads. Moreover, the stress distribution of screw and bone-screw interfaces in different models was computed in corresponding numerical mechanical models. Mechanical tests presented better screw anchoring ability with deeper screw insertion, but parameters presented no significant difference between groups with complete thread insertion. Correspondingly, higher stress values can be recorded in the model without complete thread insertion; the difference in stress values between models with complete thread insertion was relatively slight. Therefore, incomplete thread insertion triggers local stress concentration and the corresponding risk of screw loosening; completely inserting threads could effectively alleviate local stress concentration and result in the prevention of screw loosening.

## Introduction

The pedicle screw fixation system is the most widely used spinal fixation method for treating spinal trauma and degenerative and tumoral diseases (Chen et al., 2003; Karami et al., 2015). Compared with other spinal fixation methods, this method could provide better fixation stability and could be seen as the gold standard of spinal fixation (Amaritsakul et al., 2014; Ambati et al., 2015). Screw loosening is a commonly observed complication for pedicle screw-fixed patients, which triggers the loss of fixation stability and a corresponding series of issues (Bredow et al., 2016; Marie-Hardy et al., 2020). Biomechanical deterioration initially induces screw loosening. The loss of bone-screw integration is the primary pathological phenotype of screw loosening; higher stress values at the bone-screw interface cause this phenotype and corresponding screw loosening (Li et al., 2022b; Li et al., 2022c). Therefore, any risk factors that potentially trigger stress concentration on bone-screw interfaces should be considered potential risk factors for screw loosening.

Hypertrophy of articular processes is common in patients with pedicle screw fixation (Adams et al., 2000; Adams and Roughley, 2006). This change may inhibit the complete insertion of pedicle screws. Studies have reported a lower fatigue life and a higher risk of screw breakage when threads are not completely inserted into bony structures (Chen et al., 2005; Athanasakopoulos et al., 2013). Correspondingly, higher stress values of pedicle screws can be recorded in numerical models without complete thread insertion. Since the stress concentration on screws is closely related to that on the bone screw interfaces, we hypothesize that incomplete insertion of pedicle screws may also be a significant biomechanical risk factor for screw loosening; however, this has not been verified. In this study, comprehensive research combining mechanical tests and numerical simulations was performed to verify this assumption. The corresponding results should provide a theoretical foundation for optimizing fixation stability from a biomechanical perspective.

## Materials and methods

### Mechanical tests on polyurethane foams

#### Model construction

Osteoporotic polyurethane foams (Sawbones Company, United States) were used as bone substitutes due to their homogeneous structure, consistent material properties, and availability (Brasiliense et al., 2013; Amirouche et al., 2016). Since screw loosening is commonly observed in osteoporotic patients, the density of the polyurethane foam was selected to be 0.16 g/cm3 according to the standard of the American Society of Testing Materials (ASTM) protocol (Seng et al., 2019; Weidling et al., 2020). The polyurethane foam was cut to a length of 60 mm, a width of 40 mm, and a height of 50 mm. A clinically used cylindrical titanium alloy (Ti-6Al-4V) pedicle screw (with two start threads and a parallel minor diameter) (Reach-Med Company, China) was selected for this study. The outer diameter of the pedicle screw was 6.5 mm, the inner diameter was 3.5 mm, and the screw thread length was 40 mm.

**FIGURE 1:**
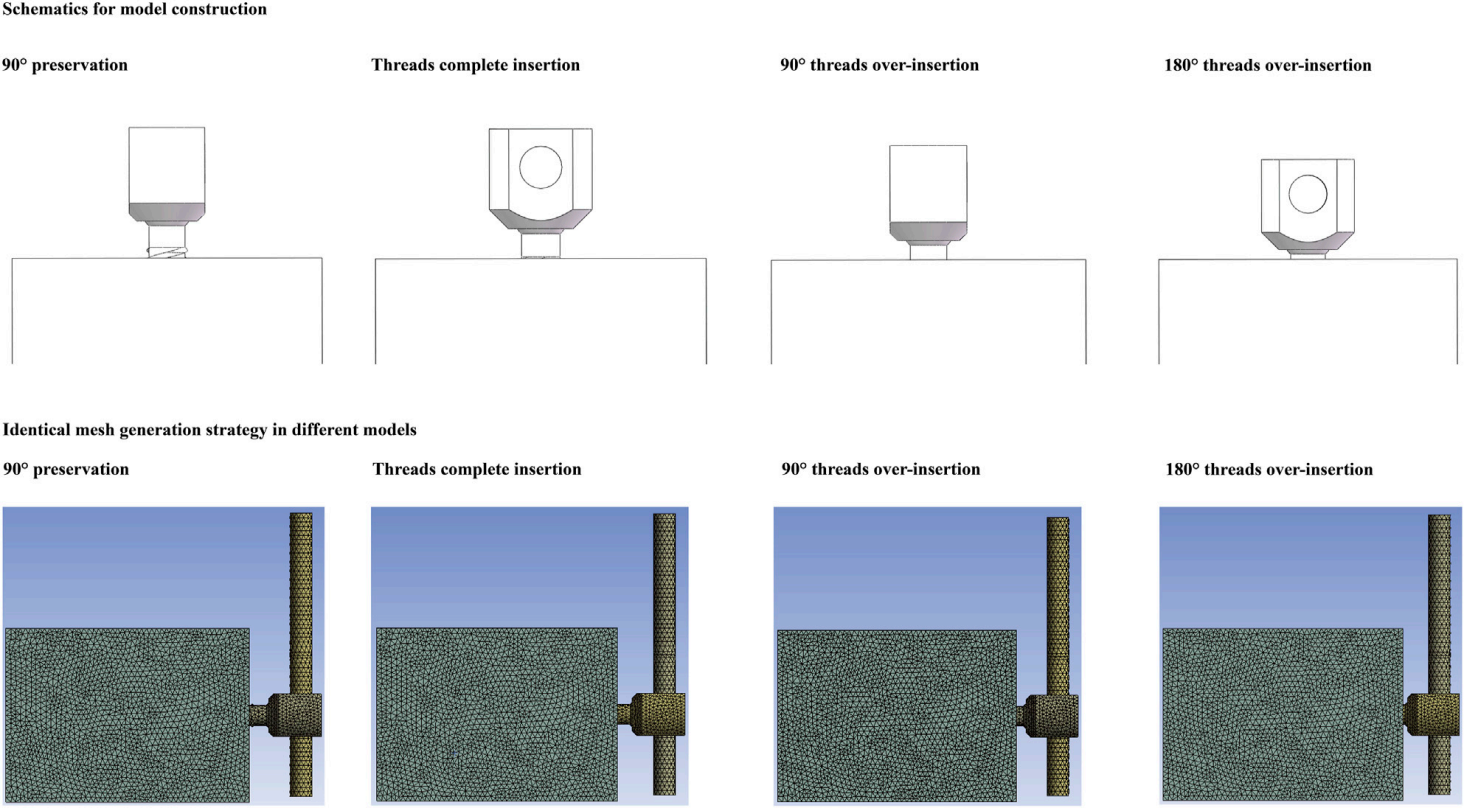
Schematic of test models with different screw insertion depths and identical mesh generation strategy in different models.

Models with four different screw insertion lengths were constructed. Models with complete thread insertion (40 mm) were considered the baseline for judging screw insertion depth. In models with incomplete screw insertion, quarter-circle threads (90°) were reserved from the test block. In contrast, in models with screw overinsertion, quarter-circle and half-circle (i.e., 90° and 180°) screws were overinserted into the test blocks, respectively (Figure 1).

## Toggle tests under different loading protocols

Toggle and pull-out tests were performed on an E3000 fatigue testing machine (Instron Company, USA). Each single test was repeated ten times in different models. Each screw, connecting rod, and nut was tested only once. Before the toggle tests, the connecting rod (6.0 mm in outer diameter and 100 mm in length) was inserted into the screw tulip and secured with nails. The axis of the rod was vertical to that of the pedicle screw, and the distance from the screw axis to the tip of the rod was set at 60 mm. For toggle testing, foam blocks were fixed in the testing machine. Each group was tested five times in different parts of the toggle tests.

Toggle tests were performed under varying cyclic loading. The pedicle screw was subjected to cyclic loading in a craniocaudal direction with stepwise increasing loads. Each pedicle screw was cyclically loaded with an initial load of ±100 N; the vertical load was increased by 25 N every 30 cycles (Brasiliense et al., 2013; Kanno et al., 2019). The instantaneous values of maximum screw displacement and the corresponding vertical load were recorded 100 times per second. Cyclic loading was terminated when screw fixation failed (the maximum screw displacement reached 1 mm). Cycle times and corresponding compressive loads at fixation failure were recorded in this procedure (Figure 1).

Moreover, when performing toggle tests under a constant load of 1*10^4^ cycles, the pedicle screw was subjected to cyclic loading in the cranio-caudal direction with a load of ±200 N. This load level was selected to simulate the physiological load of a 40-kg postmenopausal woman with osteoporosis. This is a common loading environment for screw fixation in osteoporotic patients in our country. The instantaneous values of maximum screw displacement and the corresponding vertical load were recorded 100 times per second (Pelletier et al., 2017; Wang et al., 2019b). Cyclic loading was terminated after 1*10^4^ loading cycles. The differences between the first and last displacement values were also calculated and recorded in this procedure.

### Pull-out tests

**FIGURE 2:**
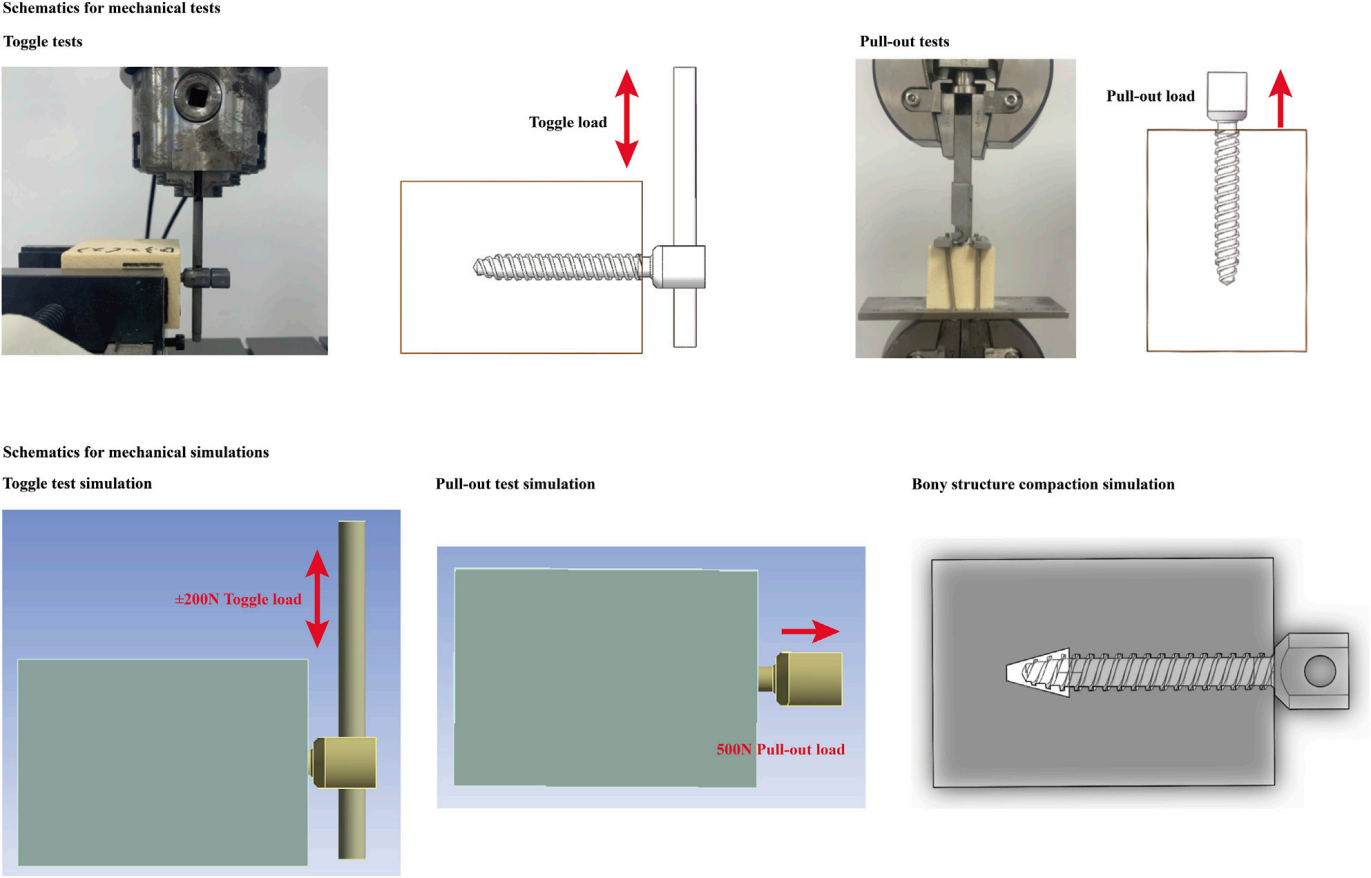
Protocols for mechanical testing and numerical simulation.

The foam-screw models in each group were subjected to pull-out tests after the toggle test with different loading protocols. In the pull-out tests, the foam was also rigidly fixed to the testing machine, and a custom-made fixture connected to the testing machine was then attached to the connecting rod. By this method, the axis of the screw was collinear with the pull-out force. All screws were pulled uniaxially at a rate of 5 mm/min until they were entirely pulled out of the foam (Chen et al., 2011; Cho et al., 2011). The pull-out strength was judged as the axial force value when a sudden decrease in the pull-out force was observed. The pull-out stiffness in different models was also recorded (Zhang et al., 2006; Yuan et al., 2014) (Figure 2).

### Statistical analyses

Mechanically tested parameters are presented as the mean ± standard deviation (Li et al., 2023; Xi et al., 2023). Statistical analyses were conducted using SPSS software. When comparing the differences between groups with different screw insertion depths, a one-way ANOVA was used for these continuous variables. A *p*-value less than 0.05 indicated a significant difference (Burger, 2023; Chatzi and Doody, 2023).

### Numerical simulations (finite element analysis)

#### Numerical model construction

The numerical model of the pedicle screw was constructed based on the outline of the screw used in the mechanical test. Therefore, the outline of the screw in the mechanical test and simulation were completely identical. Moreover, the model construction strategy, boundary conditions, and loading conditions in the numerical simulation of the toggle and pull-out tests are similar to those in the mechanical tests. To optimize computational efficiency, model simplifications were made in the numerical models. Specifically, the size of the test blocks in the numerical simulations was consistent with the mechanical tests (60*55*40 mm). The screw insertion depth was set at 40 mm. The connection between the screw tulip, the nut, and the spacer was simplified to a single model. The axis of the screw was vertical to the connecting rod, the distance between the axis of the screw and the tip of the rod was 60 mm, and the rod on the caudal side was deleted to reduce the number of elements (Li et al., 2022b; Li et al., 2022c). When defining the material properties of different components, the test blocks were set according to the official product parameter table of the saw-bone company.

Moreover, since the elastic deformation of the bony structures was present during the screw insertion process, the bony compaction (consolidation) effect caused by screw insertion was also simulated by upregulating the material properties of the surrounding bony structure around the screw tip (Figure 1). The elastic modulus of bone was assumed to be a power-law function of the density with an exponent of 2. The definition of the bony compaction region and the corresponding adjustment of its material properties were based on the same type of studies (Hsu et al., 2005; Chao et al., 2008; Travascio et al., 2017).

Moreover, the pedicle screw and connecting rod were defined as titanium alloy material (elastic modulus = 12 GPa and Poisson’s ratio = 0.31), and the definition of the test block was also performed based on the production manual of osteoporotic polyurethane foams from the Sawbone Company (elastic modulus = 23 MPa and Poisson’s ratio = 0.3). Given that the stiffness of the TC4 (120000 MPa) pedicle screw was dramatically higher than that of osteoporotic polyurethane foam (23 MPa). The deformation value of the TC4 screw was very small, so the deformation of screw can be ignored in the numerical simulaion. Therefore, the deformation of the pedicle screw was not considered in either toggle or pull-out mechanical simulations (Zhang et al., 2006; Kanno et al., 2019-2021).

### Finite element analysis under different loading protocols

#### Toggle test simulation

To ensure computational credibility, the boundary and loading conditions of the numerical simulation were kept identical to those of the toggle test. Contact types between different interfaces were defined according to the same type of study. The contact type between the screw and connecting rod was defined as “bonded”, that between bone-screw interfaces was “frictional”, and the friction coefficient was set to 0.2 (Xu et al., 2019; Takenaka et al., 2020; Li et al., 2022b). All degrees of freedom of the foam models were completely fixed, and a ±200 N load in the cranial-caudal direction was loaded on the tip of the connecting rod. To eliminate the confounding effect of mesh size, we performed a mesh convergence test on the 40 mm screw depth model. By evaluating the change in maximum equivalent stress on the pedicle screw, mesh sizes on the screw and foam were adjusted. The model was considered convergent if the change in the computed stress values was less than 3% (Ottardi et al., 2016; Li et al., 2023; Xi et al., 2023). To represent the potential risk of screw loosening, the maximum stress value of the pedicle screw and foam, the maximum shear stress and strain of the foam, and the average stress of the bone-screw interfaces were computed and recorded.

### Pull-out test simulation

The material property definition, mesh size, and contact type at the bone-screw interfaces in the pull-out test were consistent with those in the toggle test. The construction of the test block and the screw models were also consistent with the toggle test, but the connecting rod in the pull-out test was removed to reduce the number of elements (Prasad et al., 2016; Nakashima et al., 2018). The degrees of freedom of the test block were completely fixed, and a 500 N load along the axis of the pedicle screw was applied to the screw tulip. The maximum screw displacement, maximum equivalent stress on the test block, and failure volume were recorded during this procedure (Krenn et al., 2008; Bianco et al., 2017) (Figure 2).

## Results

### Mechanical test results

Overall, screw anchoring ability increased stepwise with increasing screw insertion depth. In the alternating load toggle test, the failure load of the 90° preservation and no overinsertion groups was significantly worse than that of the 180° overinsertion groups. The failure and cycle times of the 90° preservation models were significantly lower than those of the other groups, and those of the models without overinsertion were also significantly lower than those of the 180° overinsertion groups. Pull-out strength in the alternating load toggle test was also significantly lower in the 90° preservation models compared to the 180° overinsertion group. Additionally, in toggle tests with a constant load, there were no significant differences in screw anchorage parameters between the complete thread insertion groups (i.e., no overinsertion and 90° and 180° overinsertion groups). The anchoring ability of the 90° preservation group was significantly worse than that of the complete thread insertion groups (Figure 2).

### Numerically simulated results

**FIGURE 3:**
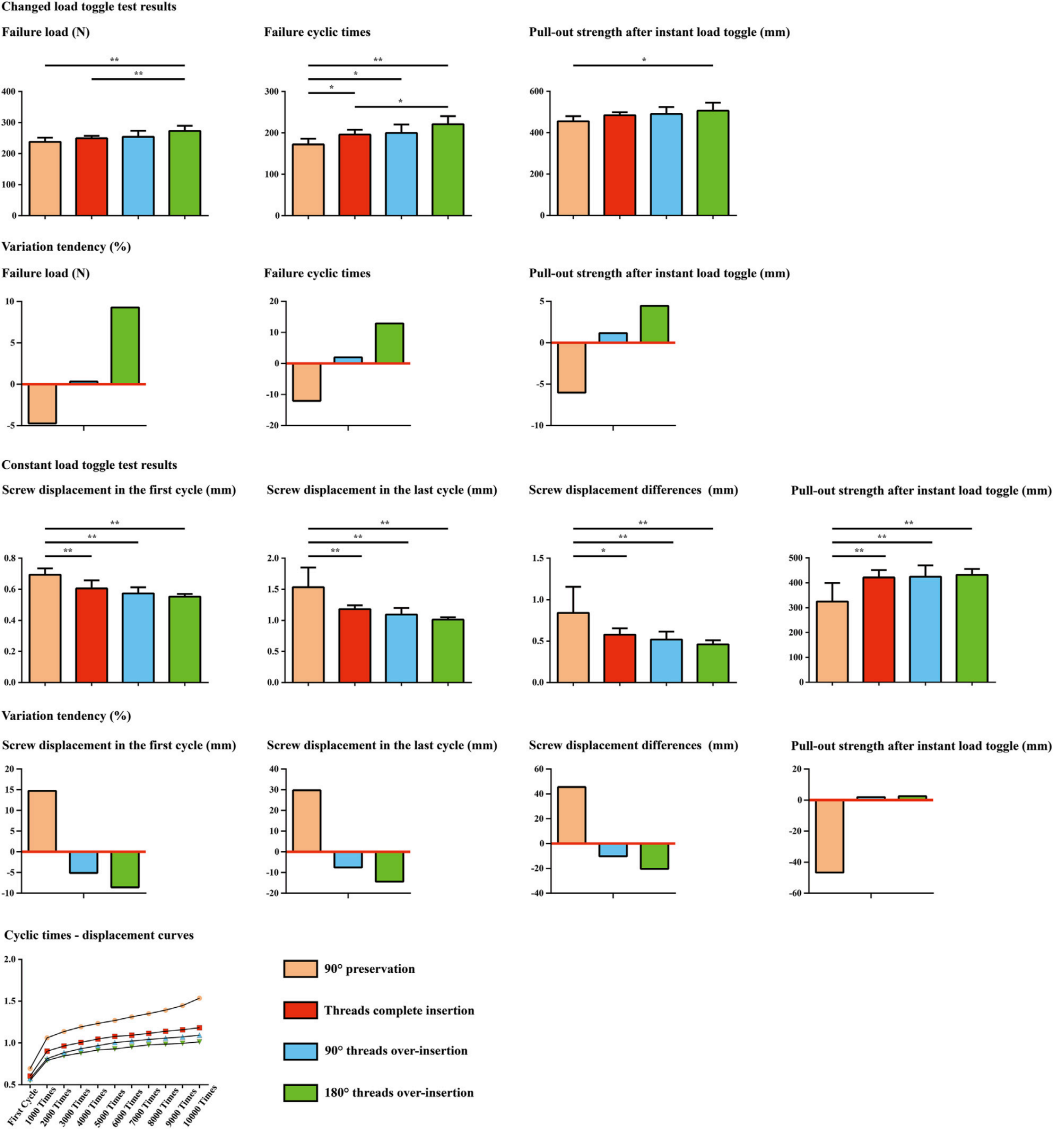
Mechanical test results in models with four different screw insertion depths.

**FIGURE 4:**
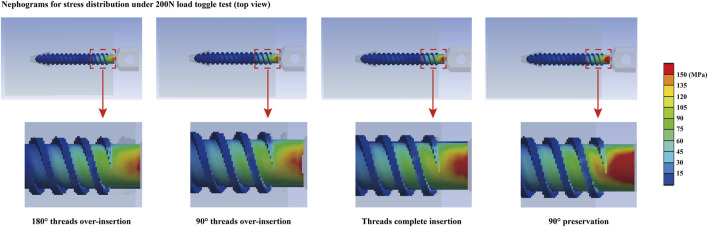
Stress distribution nephograms in models with four different screw insertion depths.

An overall consistent tendency for variation could be recorded in both toggle and pull-out mechanical simulations. Specifically, a step decrease in the maximum screw displacement stress values could be observed with a step increase in the screw insertion depth. Compared to the difference between models with and without complete screw thread insertion, the differences in stress and deformation values in models with complete thread insertion were relatively small. Detailed changes in stress and deformation values and corresponding variation percentage ratios are presented in Figures 3, 4.

## Discussion

Biomechanical mechanisms of potential risk factors for screw loosening have been identified as a stress concentration-induced complication in published studies (Yuan et al., 2014; Wu et al., 2019; Zhang et al., 2020). Although several studies have reported incorrect screw insertion and a corresponding risk of complications (Galbusera et al., 2015; Amirouche et al., 2016), the question of whether the reservation of the thread from bony structures triggers a higher risk of screw loosening remains to be addressed. Given that the selection of the screw insertion depth is a topic arising every time a screw is inserted, identifying this could provide theoretical guidance for pedicle screw insertion to biomechanically reduce the risk of screw loosening.

**FIGURE 5:**
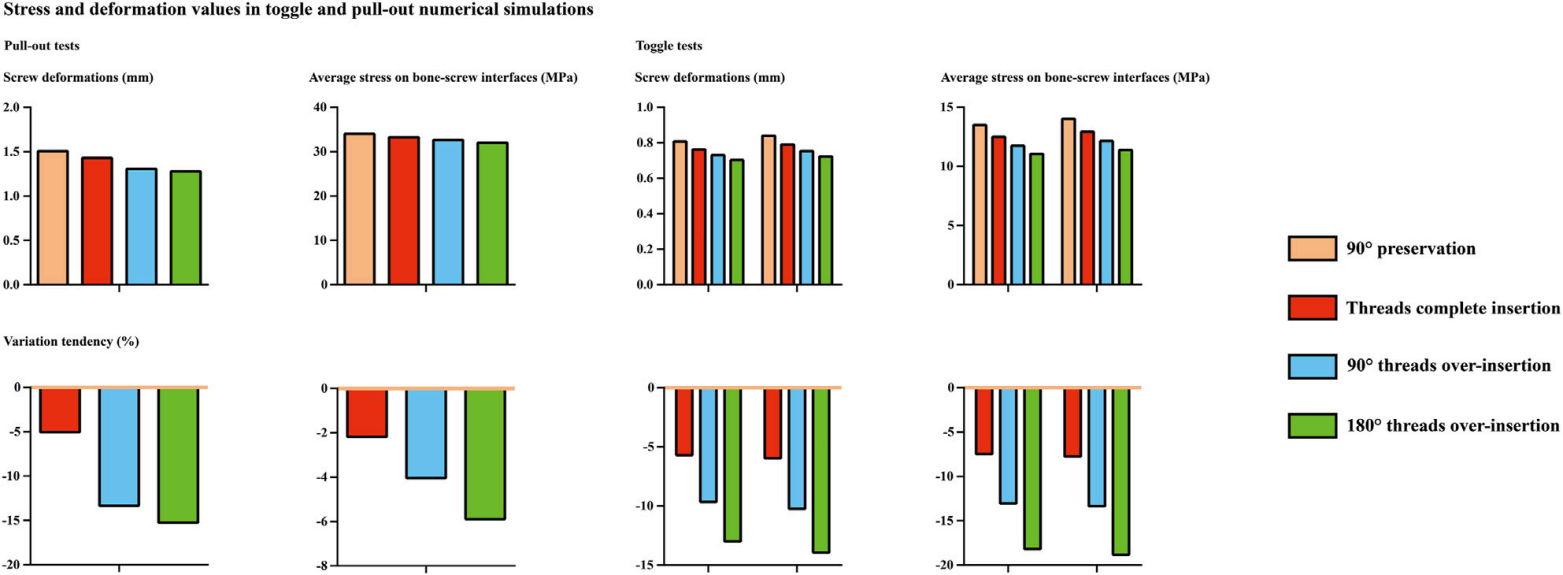
Computed stress and deformation values.

By performing comprehensive research consisting of mechanical tests and numerical simulations, this study shows that incomplete thread insertion induces local stress concentration and the corresponding risk of screw loosening; complete thread insertion could effectively alleviate the local stress concentration and optimize the screw anchoring ability. In addition, although the difference between the tested and computed results was less significant in the groups with complete thread insertion, further increasing the screw insertion depth after complete thread insertion can further optimize the screw anchoring ability. Therefore, although various factors may affect the complete insertion of the screw (e.g., hypertrophy of articular processes, occlusion of soft tissues such as the facet capsule), based on the positive correlation between screw insertion depth and anchoring ability (Figures 3, 5), complete thread insertion and even overinsertion of pedicle screws are recommended to biomechanically reduce the risk of screw loosening.

From a methodological perspective, several issues should be clarified. First, polyurethane foam models with osteoporotic bone density were selected in this study rather than vertebral bodies from specimens or any laboratory animals. Polyurethane foam can well simulate the mechanical properties of cancellous bone and has the advantages of good homogeneity (effectively eliminating the confounding effect caused by existing regional differences in cancellous bone density) (Li J. et al., 2022; Li et al., 2023) and high availability (inexpensive, and more importantly, not limited by sample sources). Therefore, this test block selection strategy may improve the feasibility and standardization of experiments, thereby increasing the credibility of this study.

However, only osteoporotic models were selected in this study. The stepwise reduction in patients’ BMD has been the most significant reason for poor screw anchoring ability by reducing the yield strength and degrading the biomechanical environment at the bone-screw interface (Xu et al., 2020; Zou et al., 2020). Biomechanical studies investigating the biomechanical effect of other risk factors (e.g., screw insertion angle, thread designs) on the risk of screw loosening also show that the incidence rate of screw loosening was consistently low in models with normal bone density, regardless of changes in other potential risk factors (Hsu et al., 2005; Galbusera et al., 2015). Therefore, osteoporotic models were selected for the current mechanical tests and corresponding numerical simulations. In addition, individual differences in the direction and size of the load applied to the pedicle screw existed in different patients but were not considered in this study (Mohammed et al., 2018). Alternatively, standard loading protocols widely used in published studies were selected in this study (Shi et al., 2012; Brasiliense et al., 2013; Wang et al., 2019a; Marie-Hardy et al., 2020; Kanno et al., 2021). The ±200 N toggle load carried by a single screw represents the old load of a patient with a body weight of 40 kg, and 500 N was selected in the pull-out test by referring to the average mechanically tested pull-out strength values in this study.

In this study, toggle tests with alternating and constant loads were performed on models with different insertion depths. Alternating load toggle tests were terminated when the maximum screw displacement value reached 1 mm because the 1 mm cavity was the standard assessment value for screw loosening. A total of 1*10^4^ times were selected in the constant load toggle tests. Screw loosening is a typical short-term complication (Galbusera et al., 2015; Amirouche et al., 2016; Ding et al., 2017). During the first 1*10^4^ cycles, the maximum screw displacement increased rapidly and remained relatively balanced (i.e., increasing the number of cycles did not significantly increase the maximum screw displacement values). Therefore, 1*10^4^ cycles are sufficient to identify the postoperative screw anchoring ability. Moreover, although the pull-out test can only directly reflect the risk of screw pull-out rather than screw loosening, this indicator has also been measured for better integration between the bone-screw interface and can optimize not only pull-out but also screw toggle strength (Wiendieck et al., 2018; Wang et al., 2019b). Consistent with this point, the current Pearson correlation analyses showed that the pull-out strength was significantly positively correlated with indicators related to the screw anchoring ability in toggle tests, and the pull-out strength was also a credible predictor when predicting the risk of screw loosening.

Mechanical tests and numerical simulations are commonly used biomechanical methods for determining screw anchoring ability (Chao et al., 2008; Solitro et al., 2022). Although mechanical tests can directly reflect screw anchoring ability by directly recording the screw displacement values in each cycle, detailed stress distribution patterns, especially at the bone-screw interfaces, cannot be directly reflected by this method. In contrast, with increasing cycle times, screw compaction on bony structures leads to higher screw displacements and results in screw loosening (Hsu et al., 2005; Boriani et al., 2018). However, this process could not be simulated in numerical models. In contrast, by comprehensively performing these two methods, the computed stress distributions can well explain the mechanism for the tested results. Also, this method can effectively optimize the reliability of the current study (Hsu et al., 2005; Chao et al., 2008).

Consisted to the same type study, direct model validation can not be performed based on the current numerical model. In finite element modeling, comparing the computed result with the mean of the test result is a common method for model validation (Li et al., 2021; Xu et al., 2022). This method is widely used in the finite element models of the intervertebral disc and facet cartilage. However, it is not suitable for screw-fixed bony structure models. Specifically, as mentioned above, bone compaction is a common phenomenon at the bone-screw interface, which can trigger an increase in bone density and elastic modulus in the compact region. Similarly, bone compaction existed not only during screw insertion but also during the toggle test process. This loading process leads to an increase in bone density around the screw, which in turn leads to an increase in screw restriction by bony structures. However, this dynamic process cannot be accurately simulated in current numerical models, which leads to the computed value of screw displacement always being larger than the tested one (this phenomenon can be observed in the current and similar studies, such as Hsu et al., 2005; Chao et al., 2008). Therefore, the above model validation method is not suitable for screw-fixed models. As an alternative, in screw anchoring ability studies where numerical simulation and mechanical tests are performed simultaneously, researchers compare the tendency of the tested and computed results, and if the trend is consistent, the numerical model is considered credible. Admittedly, this is a qualitative, rather than quantitative, approach to model validation, and screw compaction in the toggle test numerical model will be simulated in our future studies.

The neglect of cortical cells in the posterior column is an existing limitation of this study. Specifically, bony structures with irregular outlines (e.g., hypertrophied articular processes) inhibit pedicle screw insertion (Paik et al., 2012; Pelletier et al., 2017). To achieve complete screw insertion, these structures should be resected. Although cancellous bone plays a prominent role in pedicle screw anchorage, studies have also shown that this procedure damages the cortical shell of the insertion screw point and adversely affects screw anchoring ability (Paik et al., 2012; Pelletier et al., 2017; Solitro et al., 2019). The interaction between cortical damage/preservation and complete/incomplete screw insertion should be validated in future studies. Moreover, the lack of clinical evidence was due to the difficulty in accurately quantifying incomplete thread insertion during intraoperative observation and immediate postoperative imaging examination (due to the surgical field of view and titanium artifacts). Therefore, elucidating this topic through comprehensive biomechanical research has become the only feasible method.

Additionally, several factors, including changes in connecting rod materials (Athanasakopoulos et al., 2013; Boriani et al., 2020; Massey et al., 2021), screw diameters (Chao et al., 2008; Solitro et al., 2019), screw insertion orientations (Amirouche et al., 2016; Matsukawa et al., 2017; Szczodry et al., 2018), and even different screw designs (Chao et al., 2008), have been reported to affect screw anchoring ability, and interactions between these factors and incomplete thread insertion on screw anchoring ability should also be verified in our future studies. However, because all of the above parameters (i.e., rod materials, screw diameters, and orientations) were selected in this study of the most commonly used parameters in our clinical practice (the titanium alloy connecting rod was the most commonly used material, the screw axis was parallel to the fixed vertebral body, and the outer diameter was set at 6.5mm, the most commonly used diameter of a lumbar pedicle screw), we believe that these limitations will not negatively affect the credibility of the current study.

Finally, the significance of postoperative physiological and pathological processes on screw anchoring ability (Galbusera et al., 2015; Ding et al., 2017; Volz et al., 2022), including the potential osteogenic activity of different types of screw coatings (Patel et al., 2014; Li et al., 2015; Kashii et al., 2020), cannot be determined by numerical simulations and mechanical tests on polyurethane models. However, although these limitations exist, given that the mechanical tests and numerical simulations present consistent results, complete insertion of pedicle screws, especially complete thread insertion, is recommended in patients with pedicle screw fixation to optimize screw anchoring ability.

## Conclusion

Incomplete thread insertion triggers local stress concentration and higher risk of screw loosening.

## Data Availability

The original contributions presented in the study are included in the article/Supplementary Material, further inquiries can be directed to the corresponding authors.
